# Outcome of patients with perihilar cholangiocarcinoma and previous biliary instrumentation: an observational study

**DOI:** 10.1186/s12876-024-03241-8

**Published:** 2024-05-24

**Authors:** Karen T. Brown, Joanne F. Chou, Hannah B. Suchy, George I. Getrajdman, Mithat Gonen, Anne M. Covey, Lynn A. Brody, Mark A. Schattner, Michael I. D’Angelica, T. Peter Kingham, Joseph P. Erinjeri, William R. Jarnagin

**Affiliations:** 1 Department of Radiology, New York-Presbyterian Hospital, New York, NY USA; 2 Department of Epidemiology and Biostatistics, Memorial Sloan Kettering Cancer Center, New York, NY USA; 3 Department of Radiology, Memorial Sloan Kettering Cancer Center, New York, NY USA; 4 Division of Gastroenterology, Memorial Sloan Kettering Cancer Center, New York, NY USA; 5 Hepatopancreaticobiliary Surgery Division, Memorial Sloan Kettering Cancer Center, New York, NY USA; 6 Weil Medical College of Cornell University, New York, NY USA

**Keywords:** Peri-hilar cholangiocarcinoma, Bile duct cancer, Endoscopic stent, Biliary stent

## Abstract

**Background:**

To assess the outcome of previously untreated patients with perihilar cholangiocarcinoma who present to a cancer referral center with or without pre-existing trans-papillary biliary drainage.

**Methods:**

Consecutive patients with a diagnosis of perihilar cholangiocarcinoma presenting between January 1, 2013, and December 31, 2017, were identified from a prospective surgical database and by a query of the institutional database. Of 237 patients identified, 106 met inclusion criteria and were reviewed. Clinical information was obtained from the Electronic Medical Record and imaging studies were reviewed in the Picture Archiving and Communication System.

**Results:**

73 of 106 patients (69%) presenting with a new diagnosis of perihilar cholangiocarcinoma underwent trans-papillary biliary drainage (65 endoscopic and 8 percutaneous) prior to presentation at our institution. 8 of the 73 patients with trans-papillary biliary drainage (11%) presented with and 5 developed cholangitis; all 13 (18%) required subsequent intervention; none of the patients without trans-papillary biliary drainage presented with or required drainage for cholangitis (*p* = 0.008). Requiring drainage for cholangitis was more likely to delay treatment (*p* = 0.012) and portended a poorer median overall survival (13.6 months, 95%CI [4.08, not reached)] vs. 20.6 months, 95%CI [18.34, 37.51] *p* = 0.043).

**Conclusion:**

Trans-papillary biliary drainage for perihilar cholangiocarcinoma carries a risk of cholangitis and should be avoided when possible. Clinical and imaging findings of perihilar cholangiocarcinoma should prompt evaluation at a cancer referral center before any intervention. This would mitigate development of cholangitis necessitating additional drainage procedures, delaying treatment and potentially compromising survival.

## Background

Perihilar cholangiocarcinoma (PHC), a type of extrahepatic bile duct cancer, forms in the region where the left and right hepatic ducts join and is also called hilar cholangiocarcinoma or “Klatskin tumor”. It is less common in Europe & North America [1] potentially contributing to lack of familiarity with management. PHC is rarely diagnosed “incidentally” on imaging, and typically presents with clinical manifestations related to bile duct obstruction [2]. When jaundice occurs, ultrasound is usually the first imaging study performed, given low cost and widespread availability, followed by additional cross-sectional imaging when dilated ducts are identified. Magnetic resonance cholangiopancreatography (MRCP) and computed tomography (CT) angiography are complimentary studies and typically both would be obtained in patients thought to be resectable [3] Endoscopic retrograde cholangio-pancreatography (ERCP) is not part of the routine work up [4] yet is often the next step at some hospitals, frequently with concomitant stent placement across the ampulla.

In the absence of previous instrumentation, or stones, bactibilia is rarely seen in the obstructed biliary tree [5]; as a result, presentation with cholangitis is uncommon in PHC. Bilio-enteric bypass, trans-papillary drains or stents result in contamination of the biliary tree with enteric bacteria. Endoscopists have reported improved patency and lower rates of cholangitis with plastic stents that are placed above the ampulla in patients with PHC, despite sphincterotomy and retrograde injection of contrast [6]. Having seen complications related to trans-papillary biliary drainage (TPBD) derail treatment of patients with PHC, this study was undertaken to investigate the outcome of patients with a new diagnosis of PHC who had not received cancer related treatment, and presented with a pre-existing trans-papillary stent or drain, compared to those without instrumentation. The primary outcome studied was timely treatment, defined as receiving either surgery, chemotherapy, radiation, or portal vein (PV) embolization in preparation for surgery within 30 days of initial evaluation at our institution; overall survival (OS) from time of presentation was estimated.

## Methods

## Materials and methods

Consecutive untreated patients presenting to a single cancer referral center with a new diagnosis of cholangiocarcinoma during a 5-year period between January 2013 and December 2017 were identified from a prospectively maintained surgical database and query of the institutional database. This study was performed in accordance with the Declaration of Helsinki and was granted an exemption from ethics approval by the Institutional Review Board at Memorial Sloan Kettering Cancer Center; consent from the patients studied was not required given the retrospective nature of the study. Compliance with the Health Insurance Portability and Accountability Act was maintained.

235 patients with a diagnosis of cholangiocarcinoma were screened to identify those with hilar/perihilar cholangiocarcinoma. This was accomplished by review of imaging, as patients with any type of cholangiocarcinoma (intra-hepatic, hilar/perihilar and distal) shared the same diagnostic code. Imaging was reviewed by a single observer in conjunction with the official report consulting with an experienced hepatobiliary imager when necessary. Data points were obtained from the electronic medical record (EMR); imaging was reviewed in the Picture Archiving and Communications System (PACS).

129 patients were excluded, 92 based on imaging: 73 had non-hilar cholangiocarcinoma (60 intrahepatic, 8 distal hepatic/ampullary and 5 papillary) and 19 presented after resection or other treatment. Of the remaining 36 patients, 17 opted to receive care elsewhere, 15 had other cancer diagnoses, and 4 later proved to have benign disease. Thus the study group included 106 patients, 65 males (median age 67, 37–92) and 41 females (median age 70, 44–87). Cross sectional imaging including MRCP and multiphase arterial CT were reviewed to identify the tumor, establish the level of obstruction, and identify pre-existing trans-papillary stents or drainage catheters. Missing or poor-quality imaging was repeated as necessary. The clinical effectiveness of pre-existing drainage was evaluated based on the presence or absence of bile duct dilation in the stented/drained portion of the biliary tree in conjunction with the serum bilirubin after the bilirubin had reached a plateau. There were 2 situations in which drainage was considered ineffective: (1) ducts above stent were not dilated but bilirubin remained elevated and (2) biliary ducts above the stent remained dilated. The EMR of each patient was reviewed, and standard demographic data was obtained. We also recorded whether patients presented with cholangitis, jaundice, or pruritus and cases where additional percutaneous biliary drainage was required, percutaneous approach being the standard for PHC at our institution. Procedural information included indication for subsequent drainage, as well as pre- and post-procedure laboratory studies. Information regarding initial resectability, based on assessment by a Hepatobiliary surgeon, and whether the potentially resectable patient was taken to the operating room (OR) (+/- resection) or the unresectable patient was treated with chemotherapy, radiation or a combination was recorded, as well as the timing of that treatment relative to initial presentation. For calculating time-based metrics (e.g. time to treatment, overall survival) the date of diagnosis was listed as the date of first contact during the study period, despite the potential for lead-time bias, as the date of diagnosis at the outside institution was frequently impossible to assign. The primary outcome studied was timely treatment, defined as receiving either surgery, chemotherapy, radiation, or portal vein embolization in preparation for surgery within 30 days of evaluation; OS from time of presentation was estimated. 30 days was chosen as the ideal standard at our institution, where OR availability within 30 days is not an issue and patients easily receive chemotherapy or radiation appointments within that time frame.

### Statistical methods

Patients and treatment characteristics were summarized using the frequency and percentage for categorical variables, and the median and range for continuous variable. Primary outcome was receiving first treatment (surgery, chemotherapy or radiation or PV embolization) within 30 days of initial evaluation at the study institution. Fisher’s exact test and Wilcoxon rank-sum test was used to compare outcome between subgroups.

Logistic regression model was used to evaluate treatment delay, defined as undergoing surgery, chemotherapy, radiation or PV embolization more than 30 days after presentation. Multivariable logistic model was constructed, including variables that were significantly associated with outcome at *p* < 0.1 level. The final model was evaluated using Hosmer-Lemeshow “goodness of fit” test [7].

OS was calculated from the date of initial evaluation at the study institution until date of death or date of last contact. OS was estimated using Kaplan-Meier methods and compared between subgroups using log-rank test.

Using a one-month landmark analysis, OS, according to the type of first treatment (surgery vs. chemotherapy vs. radiation) was estimated. Three patients who died within 30 days of presentation were excluded.

Statistical analyses were performed using SAS Version 9.3 (SAS Institute, INC., Cary, NC, USA) or R Version 3.5.1 (R Foundation for Statistical Computing, Vienna, Austria). All *P*-values were two-sided. *P*-values of < 0.05 were considered to indicate statistical significance.

## Results

Of the 106 patients (Table 1), 73 (69%) presented with TPBD, either a stent or a catheter, and in some cases both, placed at outside institutions. There were 33 who had not been instrumented. Sixty-four of the 73 (95%) patients with TPBD had only an endoscopic stent, 1 a percutaneous stent, 4 a stent plus a drainage catheter and 4 percutaneous internal/external catheters; all devices crossed the papilla. 26 of 64 stents placed endoscopically (41%), were not effectively draining the liver at presentation; a significant predictor (*p* = 0.021) for not receiving treatment at one month. Of 73 patients with pre-existing TPBD, 8 (11%) presented to our institution with cholangitis and 29 (40%) with persistent jaundice. Of 33 patients without trans-papillary biliary drainage, none presented with cholangitis, 16 (48%) were jaundiced. Significantly more patients with TPBD presented with an elevated WBC, cholangitis or required subsequent drainage for cholangitis. Subsequent biliary drainage prior to initiating treatment was necessary in 28 (38%) of 73 patients with pre-existing TPBD. Indications included the need to decrease bilirubin for treatment in 14 (50%), cholangitis with or without hyperbilirubinemia in 13 (46%), and 1 instance of pre-operative drainage to preserve function of the liver remnant and avoid post-operative liver failure. Drainage was typically only undertaken pre-operatively when the bilirubin was 10 mg/dl or above, or in cases of a marginal liver remnant. In patients requiring chemotherapy, the goal was to reduce serum bilirubin to < 2.0 mg/dl. In 33 patients without previous TPBD, 17 patients (51%) required subsequent drainage to allow for chemotherapy, all with hyperbilirubinemia, 6 of these were also pruritic. 75 of the 106-patient cohort (71%) were thought to have resectable disease at presentation, 50 of 73 patients (68%) with TPBD and 25 of 33 (76%) without. 90% of those thought to be resectable in each group were taken to the OR. 22 (29%) did not undergo resection. Seven patients were never taken to the OR, 5 with previous TPBD and 2 without; All 5 with pre-existing TPBD had recurrent cholangitis, 1 had a complication related to portal vein embolization and the other declined surgery. The other 15 patients proved to have metastasis (10), vascular involvement (4) or extensive local spread (1) at the time of surgery that precluded resection.

**Table 1:** Patient characteristics according to previous TPBD or not

Characteristic	Overall, *N* = 106	NO, *N* = 33	YES, *N* = 73	*p*-value
**Age at diagnosis, years, median (IQR)**	68 (62, 76)	67 (60, 77)	69 (62, 75)	0.4
**Gender**				> 0.9
Female	41 (39%)	13 (39%)	28 (38%)	
Male	65 (61%)	20 (61%)	45 (62%)	
**WBC at presentation, median (IQR)**	7.4 (5.6, 9.4)	6.6 (5.1, 7.7)	8.0 (6.1, 10.2)	0.003
**Bilirubin level at presentation, median (IQR)**	5 (1, 12)	9 (2, 13)	3 (1, 11)	0.032
**Presented with cholangitis**				0.055
No	98 (92%)	33 (100%)	65 (89%)	
Yes	8 (7.5%)	0 (0%)	8 (11%)	
**Presented with jaundice**				0.4
No	61 (58%)	17 (52%)	44 (60%)	
Yes	45 (42%)	16 (48%)	29 (40%)	
**Presented with either cholangitis or jaundice**				0.7
No	59 (56%)	17 (52%)	42 (58%)	
Yes	47 (44%)	16 (48%)	31 (42%)	
**Presented with or drained for cholangitis**				0.005
No	91 (86%)	33 (100%)	58 (79%)	
Yes	15 (14%)	0 (0%)	15 (21%)	
IR procedure for cholangitis*				0.008
No	93 (88%)	33 (100%)	60 (82%)	
Yes	13 (12%)	0 (0%)	13 (18%)	
IR procedure for Hyperbilirubinemia*				0.077
No	69 (65%)	17 (52%)	52 (71%)	
Yes	37 (35%)	16 (48%)	21 (29%)	
IR procedure for pruritis *				0.067
No	96 (91%)	27 (82%)	69 (95%)	
Yes	10 (9.4%)	6 (18%)	4 (5.5%)	
IR procedure for pre-operative *				0.022
No	93 (88%)	25 (76%)	68 (93%)	
Yes	13 (12%)	8 (24%)	5 (6.8%)	
**Resectable or not**				0.5
No	31 (29%)	8 (24%)	23 (32%)	
Yes	75 (71%)	25 (76%)	50 (68%)	

^*^IR procedure prior to initiation of any treatment; TPBD = Trans-papillary biliary drainage; IQR = Interquartile range. WBC = White blood cells.

Table  2 illustrates the univariate logistic regression analysis. Presenting with jaundice, leukocytosis, cholangitis (or cholangitis as the indication for a subsequent drainage procedure) or a stent not effectively draining the liver were associated with delay in treatment beyond 30 days of presentation. Multivariate logistic regression analysis showed that patients presenting with jaundice had approximately a 3-fold risk of treatment delay compared to patients who were not jaundiced (OR: 3.41 (95%CI:1.38–8.82). In addition, although not statistically significant, patients with cholangitis (OR 4.4, 95% CI 0.97–31.7, *p* = 0.081) or with a non-functioning stent at presentation (OR 2.14, 95% CI 0.65–07.45, *p* = 0.22) were also less likely to be treated within 30 days (data not shown).

**Table 2:** Factors associated with delay in receiving treatment*

Characteristics	Not treated within 30 days, *N* = 56^1^	Treated within 30 days, *N* = 50^1^	OR^1^	95% CI^1^	*p*-value
**Age at diagnosis, median (IQR)**	68 (62, 78)	69 (60, 74)	1.03	0.99, 1.06	0.12
**Gender**					
Female	24 (43%)	17 (34%)	—	—	
Male	32 (57%)	33 (66%)	0.69	0.31, 1.51	0.4
**WBC at presentation, median (IQR)**	7.9 (6.6, 10.1)	6.6 (5.2, 8.2)	1.21	1.06, 1.40	0.009
**Bilirubin level at presentation, median (IQR)**	7 (1, 12)	2 (1, 9)	1.04	0.99, 1.10	0.14
**Presented with cholangitis**					
No	49 (88%)	49 (98%)			
Yes	7 (13%)	1 (2.0%)			
**Presented with jaundice**					
No	24 (43%)	37 (74%)	—	—	
Yes	32 (57%)	13 (26%)	3.79	1.69, 8.88	0.002
Presented or drained for cholangitis*			7.26	1.87, 48.1	0.012
0	43 (77%)	48 (96%)			
1	13 (23%)	2 (4.0%)			
**STENT status**					
No prior STENT	14 (25%)	19 (38%)	—	—	
Prior STENT/not draining liver	19 (34%)	7 (14%)	3.68	1.26, 11.7	0.021
Prior STENT/draining liver	23 (41%)	24 (48%)	1.30	0.53, 3.22	0.6

* Treatment was defined as receiving surgery, chemotherapy, radiation, PV embolization within 30 days of presentation

^* 1^ Univariate logistic regression model estimating the odds ratio of treatment delayed ; OR = Odds ratio CI = confidence interval

**Fig. 1:**
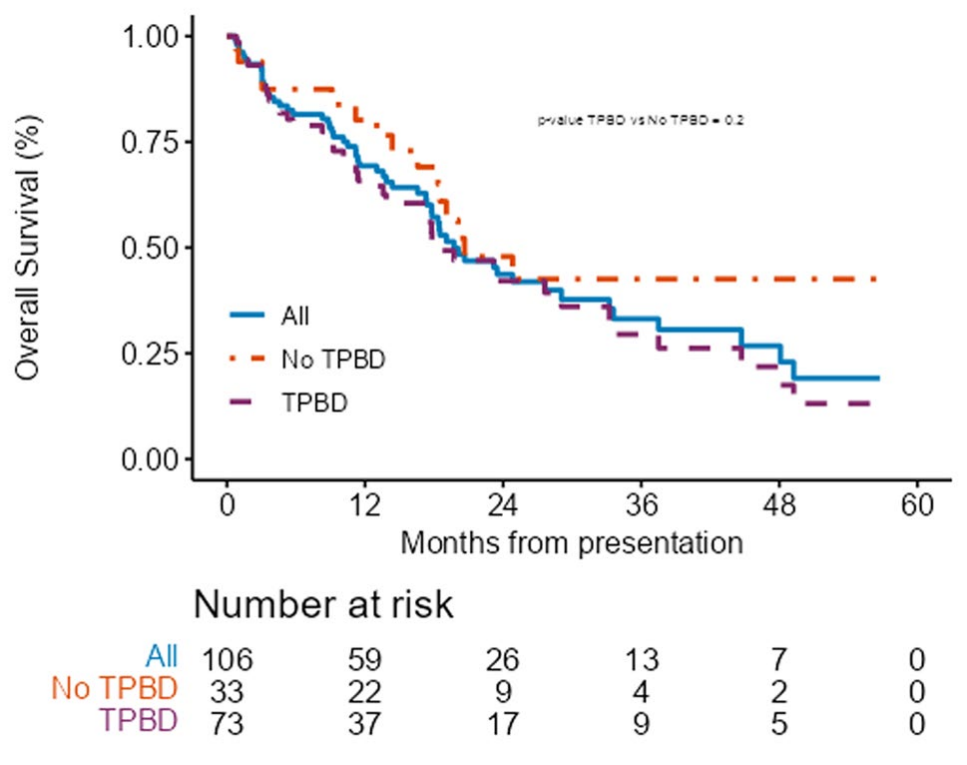
Overall survival for entire group (solid blue line), patients who presented without a TPBD (orange dotted line) and those that presented with TPBD (purple dashed line)

**Fig. 2:**
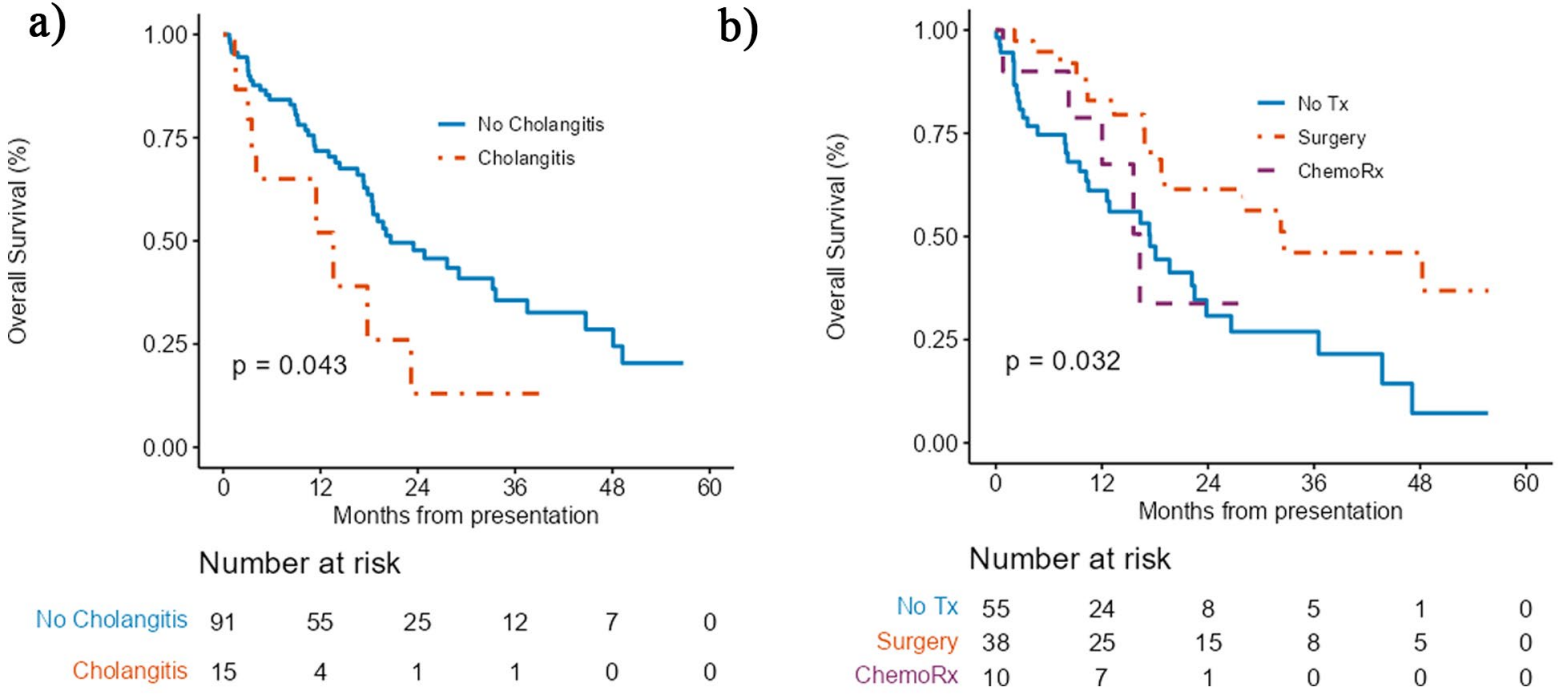
**a** Overall survival of patients presenting with (orange line) or without (blue line) cholangitis, **b** Overall survival of patients alive at 30 days by treatment group; No treatment (blue line), Surgery (orange line) and chemotherapy (purple line), p value is a global comparison of all 3 groups

Median follow-up among survivors was 15.3 months (range 2–56) and median OS for the entire cohort was 19.7 months [95%CI (17.8–33.2)]. OS in the group without TPBD was 20.6 months [95%CI (18.34 – not reached (NR))] compared to 18.4 months [95%CI (13.84–33.6)] for those with TPBD (ns) (Fig. 1). The 1-year OS was 80.17% [95%CI (67.06–95.84)] without TPBD, and 64.54% [95%CI (53.99–77.16)] for those with TPBD. Median OS for those with cholangitis at presentation was 13.6 months, [95%CI 4.08 - NA] while it was 20.6months [95%CI 18.34–37.51] for those without cholangitis *p* = 0.043 (Fig. 2a). Among the patients alive at 1 month, OS for those not treated was 17.4 months (95% CI 10.47–26.61), for those receiving chemotherapy 16.3 months [95% CI 12 – NR] and for those who were resected, 32.6 months [95% CI 19.1 – NR] (Fig. 2b).

## Discussion

Unlike distal bile duct cancers, PHC often extends to involve the confluence of the biliary tree, and may involve the sectoral or segmental ducts. In this instance, the entire biliary tree cannot be drained with a single stent. While the entire liver does not need to be drained for relief of jaundice, the larger percentage of liver drained, the more likely it is that jaundice will resolve [8]. This depends on having portal venous flow to the region that is drained [9]. Tumors that arise in either right or left hepatic duct may involve the ipsilateral portal vein. When it is not possible to drain the entire system with one stent, and there is unilateral portal vein compromise, clinically successful biliary drainage requires careful pre-procedure planning, targeting the region of the liver with preserved portal blood flow. When drainage is performed pre-operatively the future liver remnant (FLR) should be targeted. Whether pre-operative drainage is necessary remains controversial, particularly in patients with FLR > 50% [3, 10].

Advanced endoscopic techniques have improved the technical success of trans-ampullary stent placement performed for relief of jaundice. However, retrograde injection of contrast material and concomitant sphincterotomy, results in contamination of the biliary tree with enteric bacteria. Bacterial contamination of a portion of the biliary tree that remains undrained will result in development of cholangitis. Despite improvement in endoscopic techniques, a high risk of inadequate pre-operative endoscopic drainage has been shown in a recent study [11]. In this report, 38% of patients with operable PHC required additional percutaneous procedures after ERCP [11] due to therapeutic failure, cholangitis, technical failure and/or insufficient drainage of the FLR. Stents may be inserted below the level of obstruction or positioned within a region of the liver without portal venous inflow; in either situation the stent is ineffective, jaundice does not resolve, and cholangitis may develop. As stated in the 2013 Asia-Pacific consensus recommendations for management of hilar cholangiocarcinoma “The advantage of percutaneous approach is the precise lobar selection for drainage” [12]. Targeting is critical; incomplete drainage of the FLR in patients undergoing resection for perihilar cholangiocarcinoma is associated with post-operative mortality, particularly with small or intermediate FLRs [10]; this risk is greater when preoperative cholangitis develops. The percutaneous approach allows for accurate targeting and for drainage without compromising the ampulla and introducing enteric bacteria, by ensuring the stent or catheter does not enter the duodenum.

Pre-existing TPBD is a risk factor for presenting with or requiring treatment for cholangitis in the current study. Cholangitis, and presenting with ineffective drainage, occurred in 36% of patients with TPBD, factors that lowered the probability of treatment within one month. Notably, although 26 patients (36%) were not effectively drained at presentation, 31 patients (42%) with TPBD presented with jaundice or cholangitis. Persistent jaundice in the face of “effective drainage” occurs when the TPBD drains the part of the liver into which it was placed, but either that part is non-functional liver without portal vein flow or the volume of liver drained is too small, perhaps a single segment. In either case, jaundice persists. Cholangitis may occur when the TPBD effectively drains one portion of liver, if other parts of biliary tree have been contaminated and remain undrained. Presentation with cholangitis, or subsequent drainage for cholangitis, are both associated with poorer overall survival. 18% of patients with TPBD required subsequent percutaneous intervention for cholangitis prior to treatment; none of the patients without TPBD experienced cholangitis, similar to findings by Wiggers et al., who noted that none of 58 patients without preoperative drainage developed cholangitis [10]. The ability to target the largest functional portion of the liver and provide drainage without crossing the ampulla supports a percutaneous approach [3].

A study of 240 patients from a consortium of 10 Academic Medical Centers in the United States found no difference in long-term oncologic outcome, including disease specific survival (DSS) and recurrence free survival (RFS), following presurgical percutaneous transhepatic biliary drainage (PTBD) and endoscopic biliary drainage (EBD) in patients with PHC; this was a retrospective study of patients selected from a multi-institutional database who had undergone R1 or R0 resection for hilar cholangiocarcinoma [13]. These patients had already cleared preoperative hurdles, including their drainage procedure, and “made it to treatment” and, as a result, this study cannot discriminate between the utility of percutaneous or endoscopic drainage in the group from time of presentation. There was no comment on whether there was a subgroup of patients with cholangitis who never made it to surgery, as was the case in 7% of patients initially thought resectable in the current study. The consortium study found no difference in DSS or RFS despite the PTBD patients having more advanced disease. Incidentally, the pattern of recurrence in that study was similar between the 2 groups, refuting previous claims of a high incidence of track seeding occurring after percutaneous drainage procedures [14].

The debate surrounding percutaneous versus endoscopic stent placement for PHC has been ongoing, with papers supporting one or the other technique. PHC is more common in Asia than in the Europe and North America, and in 2013 the Asia-Pacific consensus found percutaneous stenting superior to endoscopic for managing PHC, citing a higher success rate, lower risk of cholangitis and similar complication rates and survival [12]. In 2015 Zhao et al. performed a meta-analysis comparing percutaneous vs. endoscopic biliary drainage for malignant biliary obstruction [15]. 554 patients were studied in 6 different trials between 2002 and 2013. 503 (90.5%) of these patients had PHC and 54 had pancreaticobiliary malignancy or metastatic nodal disease as cause of obstruction, the majority clearly had hilar obstruction. After excluding 2 studies suggested to be outliers, heterogeneity decreased markedly, and the study supported better therapeutic success with a lower incidence of cholangitis associated with the percutaneous approach. The overall complication rate, incidence of pancreatitis and 30-day mortality of the two procedures was similar.

Conversely, a randomized trial of pre-operative PTBD versus EBD reported in 2018 by Coelen et al. strongly recommended against percutaneous drainage for cholangiocarcinoma [16]. This trial was prematurely stopped because of the high overall mortality (11/27, 41%) in the PTBD group compared to the EBD group (3/27, 11%), including 3 of 27 (11%) PTBD patients dying pre-operatively, despite a similar number of severe complications occurring in each group following drainage. This is in stark contrast to previous studies that have demonstrated the opposite findings, including a meta-analysis from 2017 by Al Mahjoub et al. that reported significantly lower risk of procedure related morbidity, 44.3% vs. 26.5%, in 275 patients drained endoscopically vs. 158 with percutaneous drainage (*p* = 0.0009) [17]. In this meta-analysis, the rate of conversion from one procedure to the other was significantly lower in the percutaneous group (*p* < 0.00001) and the only patients who developed pancreatitis were in the endoscopic group. The cholangitis rate was significantly lower in the percutaneous group compared to the endoscopic group, 7.6% vs. 33.8% respectively (*p* < 0.00001).

The findings of the Coelen paper are difficult to interpret, and the 11% procedural mortality rate in the PBD group prior to surgery is unusual; higher than the 3.3% threshold endorsed by Society of Interventional Radiology Standards[20]. The authors have recently performed a pilot trial of primary percutaneous stenting (PPS), keeping the stent above the ampulla, with results supporting the development of a randomized controlled trial (RCT) to compare PPS versus endoscopic stenting. Such a trial would be most welcome and likely to address many of the questions raised by their 2018 RCT.

The current study has several limitations common to most retrospective analyses of patients with uncommon tumors. Being a single institution study of slightly more that 100 patients, institutional paradigms may result in the introduction of treatment bias that might not be applicable to other institutions or patient populations. The lack of difference in survival between treatment with chemotherapy compared with no treatment is surprising and potentially related to the small number of patients in the chemotherapy group. Both groups had a median survival of over one year despite shorter survivals of 12.5 months for chemotherapy and 2.9 months for untreated patients cited in a descriptive review by Waseem and Tushar [18]. Survival of those treated surgically is in keeping with median survival reported by others [18].

This report supports the concept that patients with PHC who undergo TPBD early in their work-up, whether endoscopic or percutaneous, fare worse than patients who present without drainage. This is thought to be secondary to introducing enteric bacteria by having the device across the ampulla, coupled with drainage of insufficient volume of liver or of parenchyma lacking portal blood flow. TPBD was demonstrated to result in need for further drainage related to jaundice or cholangitis in 38% of patients, the same percentage reported by Wiggers et al [11]. Pre-existing TPBD was associated with delay in treatment and poorer overall survival. Patients presenting with painless jaundice and thought to have bile duct malignancy based on clinical and imaging characteristics would benefit from evaluation by an experienced hepatobiliary surgeon at a high-volume center before their biliary tree is instrumented. ERCP to establish a tissue diagnosis, without regard to whether the tumor is resectable, should be avoided. Zhou H-F et al. have shown that prior surgical or endoscopic interventions in the biliary tree, and high bile duct obstruction, are independent predictors for early biliary infectious complications following metal stent placement [19]. The development of cholangitis early in the course of evaluation, as demonstrated, can compromise and/or delay definitive therapy, as recurrent cholangitis precludes surgery, administration of chemotherapy or radiation therapy and is associated with poorer survival. When biliary drainage is indicated, and obstruction is at the hepatic hilus isolating at least the right and left ducts, drainage should be targeted to provide drainage of the largest volume of liver parenchyma. Accurate targeting is best accomplished percutaneously. Surgical candidates may have an external drain placed across the hilar obstruction, ending in the common bile duct above the ampulla, avoiding contamination of the biliary tree with enteric organisms, as occurs when a catheter crosses the ampulla [3]. This is essential in management of PHC, given that cholangitis is known to be a significant predictor of post-operative mortality [10]. Unresectable patients may have a primary self-expanding metal stent (SEMS) placed percutaneously, keeping the distal end of the stent above the ampulla, thus preserving sphincter function, and excluding enteric organisms, diminishing the risk of subsequent cholangitis.

## Conclusion

As most patients with PHC are asymptomatic at initial presentation, goals of treatment should be clearly defined prior to any procedure that results in inoculation of the biliary tree with enteric bacteria. When biliary drainage is indicated, placement of stents or drains across the ampulla appears to result in less favorable outcome.

## Data Availability

The datasets generated and/or analyzed during the current study are not publicly available as they are maintained in a HIPAA compliant database at the study institution. HIPAA compliant data can be made available from the corresponding author on reasonable request.

## Abbreviations

PHC: Perihilar cholangiocarcinoma
MRCP: Widespread availability, followed by additional cross-sectional imaging when dilated ducts are identified. Magnetic resonance cholangiopancreatography
CT: Computed tomography
ERCP: Endoscopic retrograde cholangio-pancreatography
TPBD: Trans-papillary biliary drainage
PV: Portal vein
OS: Overall survival
EMR: Electronic medical record
PACS: Picture archiving and communications system
OR: Operating room
FLR: Future liver remnant
DSS: Disease specific survival
RFS: Recurrence free survival
PTBD: Percutaneous transhepatic biliary drainage
EBD: Endoscopic biliary drainage
PPS: Primary percutaneous stenting
RCT: Randomized controlled trial
